# Sustainable Production of Lactic Acid from Cellulose Using Au/W-ZnO Catalysts

**DOI:** 10.3390/polym15214235

**Published:** 2023-10-26

**Authors:** Mingyu Guo, Chengfeng Zhou, Yuandong Cui, Wei Jiang, Guangting Han, Zhan Jiang, Haoxi Ben, Xiaoli Yang

**Affiliations:** State Key Laboratory of Bio-Fibers and Eco-Textiles, College of Textiles and Clothing, Qingdao University, Qingdao 266071, China; 2021020773@qdu.edu.cn (M.G.); chengfengzhou@qdu.edu.cn (C.Z.); cydzxy923@163.com (Y.C.); weijiangqd@qdu.edu.cn (W.J.); kychgt@163.com (G.H.)

**Keywords:** cellulose conversion, lactic acid, reaction mechanism, rate-limiting step

## Abstract

The catalytic conversion of cellulose to lactic acid (LA) has garnered significant attention in recent years due to the potential of cellulose as a renewable and sustainable biomass feedstock. Here, a series of Au/W-ZnO catalysts were synthesized and employed to transform cellulose into LA. Through the optimization of reaction parameters and catalyst compositions, we achieved complete cellulose conversion with a selectivity of 54.6% toward LA over Au/W-ZnO at 245 °C for 4 h. This catalyst system also proved effective at converting cotton and kenaf fibers. Structural and chemical characterizations revealed that the synergistic effect of W, ZnO, and Au facilitated mesoporous architecture generation and the establishment of an adequate acidic environment. The catalytic process proceeded through the hydrolysis of cellulose to glucose, isomerization to fructose, and its subsequent conversion to LA, with glucose isomerization identified as the rate-limiting step. These findings provide valuable insights for developing high-performance catalytic systems to convert cellulose.

## 1. Introduction

As the enormous consumption and depletion of fossil fuels has led to an escalating environmental and energy crisis, producing industrial chemicals from renewable biomass has attracted considerable attention due to its sustainability [1]. Cellulose, as the most abundant biomass resource, is a linear polymer compound consisting of D-glucopyranose rings linked by β-(1,4)-glucoside bonds. It can be transformed into high-value chemicals such as lactic acid (LA), formic acid, acetic acid, ethylene glycol, and propylene glycol [2,3]. Among these chemicals, LA is a promising platform compound that can generate various derivatives such as acrylic acid, propylene glycol, and pyruvic acid. Moreover, LA can be utilized to produce biodegradable polymers [4,5]. Currently, more than 90% of LA is derived from fermentation processes that consume corn starch and, thus, exacerbate the food shortage issue. In addition, the production cycle is long, usually ranging from a few days to a few weeks, which increases time costs [6,7]. However, chemical methods can use a wide range of raw lignocellulosic biomass, greatly reducing costs and providing technical support for the country’s circular economy. In addition, the production cycle is short and can be completed in a few hours, which greatly reduces the time cost. Therefore, the catalytic conversion of cellulose to LA is an alternative method with important research significance [8].

Despite this approach’s potential, cellulose conversion is limited by the difficulty of cellulose hydrolysis, and various side reactions can occur during the cellulose conversion process, resulting in the low selectivity of LA. Some researchers have explored homogeneous acid-base catalysts to improve cellulose conversion and LA selectivity. For instance, Epane et al. applied microwave-assisted alumina to convert glucose under alkaline conditions, achieving a high LA yield of 75% [9]. Zhang et al. fabricated the Zn/Ni/C catalyst and used a NaOH aqueous solution to obtain a LA yield of 42% [10]. However, liquid alkali catalysts pose challenges, such as equipment corrosion and catalyst recovery difficulties. Alternatively, researchers have discovered that adding transition metal ions can improve LA yield because the vacant orbitals provided by transition metal ions can coordinate and activate alcohol hydroxyl hydrogen in cellulose [11]. Bicker et al. utilized Zn^2+^ to convert sucrose to lactic acid under hydrothermal conditions at 300 °C and observed a LA yield of 42% [12]. Nevertheless, metal salt catalysts still encounter challenges in product separation.

In this regard, solid acid catalysts offer several advantages compared to homogeneous catalysts, such as easy product separation after a reaction, a high recovery and reuse rate, non-toxicity, and low environmental pollution. It can also be analyzed for reaction pathways through deuterium exchange [13]. For instance, a ZrO_2_ catalyst in a neutral aqueous solution can facilitate the catalytic conversion of xylose or xylan to lactic acid, with a yield of 42% at 200 °C and 2.4 MPa N_2_ for 40 min. The sites on the surface of ZrO_2_ are conducive to the reverse aldol condensation reaction of xylose and promote the formation of lactic acid [14]. Similarly, ZnO, a low-cost metal oxide with rich L acidic sites, can convert xylose into LA [15]. Jin et al. used commercial ZnO as an efficient catalyst for converting glucose into LA under hydrothermal conditions, which obtained a LA yield of 45%. The ZnO catalyst could be reused five times without losing its catalytic activity [16].

In addition, tungstate solid acid catalysts have strong acidity, green environmental protection characteristics, and exemplary performance in preparing lactic acid from cellulose. Nguyen et al. used ZrW to convert cellulose into LA, obtaining a yield of 45.1%. The activity of ZrW was attributed to WO_X_ dispersed on its surface [17]. Tungsten oxides or carbides can assist in breaking the C-C bond of cellulose into intermediates [18]. Noble metal catalysts have also shown high catalytic activity for converting glycerol to lactic acid under anaerobic conditions [19]. Lakshmanan et al. found that Au/CeO_2_ catalysts can efficiently catalyze the selective oxidation of glycerol to lactic acid at atmospheric pressure with higher selectivity [20]. Xu et al. used AuPd/TiO_2_ to oxidize glycerol to lactic acid, obtaining a yield of 56.8% [21].

In this work, we employed ZnO as the support, W as the promoter, and loaded Au metal to prepare a series of catalysts for the conversion of cellulose to LA. Recycle tests were conducted to study the stability of the catalyst under hydrothermal conditions. Furthermore, cotton fiber and kenaf fiber were utilized to test the performance of the catalysts for natural lignocellulosic feedstocks. The structural properties of these catalysts were investigated, exploring the roles of separate components. Additionally, based on the designed experiments, a plausible reaction route for cellulose conversion is proposed.

## 2. Experimental Section

### 2.1. Materials and Catalyst Preparation

Trimesic acid, zinc acetate dihydrate, sodium tungstate, lactic acid (LA), formic acid (FA), acetic acid (AA), acetone alcohol (Ac), propylene glycol (PG), dihydroxyacetone (DHA), propionaldehyde (PLY), N,N-dimethylformamide (DMF), glucose, fructose, sodium borohydride, chloroauric acid and cellulose powder were all purchased from Shanghai Aladdin Biochemical Technology Co., Ltd. (Shanghai, China). The cotton and kenaf samples were collected from Xinjiang Province, China. Cotton samples were stored under dry conditions. Before the experiment, the skin was separated from the nucleus, cut to a length of about 1–2 cm, and stored in dry conditions after air drying.

The supports were first synthesized. A total of 1.26 g of H_3_BTC and 1.1 g of Zn(Ac)_2_·2H_2_O was dissolved into 50 mL of DMF; then, 0.329 g of sodium tungstate was added with continuous stirring for 2 h. Subsequently, the solid sample was obtained via filtration, washed with deionized water, and dried at 60 °C overnight, followed by calcination under an N_2_ atmosphere at 550 °C for 3 h, named W-ZnO. Similarly, the ZnO support was prepared with the same method but without adding sodium tungstate. In addition, the W-ZnO sample was stirred in 1 mol L^−1^ of a hydrochloric acid solution for 2 or 4 h, respectively, to decrease the ZnO content. They were then labeled as W-ZnO-2 or W-ZnO-4.

Next, metal Au was impregnated over the supports and was in situ reduced with NaBH_4_. Specifically, 0.2 g of the support was dispersed into 50 mL of deionized water, then 0.63 mL of chloroauric acid (0.976 g_Au_ L^−1^) was added and stirred for 30 min. In total, 25 mg of NaBH_4_ was added to the above mixture with continuous stirring in the ice-water bath. After 30 min of the reaction, the final products were recovered via filtration, then washed with deionized water and dried at 60 °C for 6 h in the vacuum oven. The samples were named Au/W-ZnO, Au/ZnO, Au/W-ZnO-2, and Au/W-ZnO-4.

### 2.2. Activity Measurements

In a typical reaction, 50 mg of the catalyst, 100 mg of cellulose powder, and 30 mL of H_2_O were added into a 100 mL autoclave with Teflon lining. After ultrasonication for 10 min and purging with N_2_ several times, the autoclave was charged with 2.0 MPa N_2_ at room temperature, then heated to 245 °C and kept for 4 h with continuous stirring. Generally, the reactions were conducted under the conditions of 2.0 MPa N_2_, 245 °C for 4 h if no special instruction existed. For the recycling test, catalysts were recovered using centrifugation after each reaction, washed with water several times, and dried at 60 °C for 8 h in the vacuum oven.

The liquid samples were identified and quantified using high-performance liquid chromatography (HPLC, Agilent 1260, Shanghai, China) with an 87-H column and a refractive index detector (RID). The mobile phase comprised 2 mmol L^−1^ of a H_2_SO_4_ aqueous solution with a rate of 0.6 mL min^−1^.

The conversion of cellulose was calculated according to the mass change, while the yields of products were estimated based on carbon.

X_cellulose_ (%) = (m_raw cellulose_ − m_residual cellulose_)/m_raw cellulose_ × 100

Y_product_ (%) = mol_carbon in a specific product_/mol_carbon in raw cellulose_ × 100

All the tests were repeated three times to ensure the reliability of the results, and the error was less than 5%.

### 2.3. Catalyst Characterizations

The element contents in these catalysts were determined using the Inductive-Coupled Plasma Emission Spectrometer (ICP, PerkinElmer, Waltham, MA, USA). The samples were treated under a vacuum at 120 °C for 5 h before analysis. The composition and crystal structure of the samples were obtained using X-ray diffraction (XRD) measurements equipped with Cu Kα radiation. The surface area of the samples was determined using N_2_ physical adsorption–desorption isotherms and an ASAP 2460 Quantachrome instrument (Microtrac, Osaka, Japan). The morphology and elemental mapping of the catalysts were observed on a JSM-6390LV scanning electron microscopy (SEM, Carl Zeiss AG, Jena, Germany) equipped with an energy-dispersive spectroscopy analyzer (EDS). The catalysts’ metal particle size and lattice stripe spacing were observed using a JEM 2100F transmission electron microscope (TEM, JEOL, Tokyo, Japan). The X-ray photoelectron spectra (XPS) of elements were measured using a Thermo ESCALAB 250Xi apparatus with Al Kα as the X-ray source (Thermo Fisher, Brno-Černovice, Czech Republic). The binding energy of the C1s peak at 284.8 eV was used as a reference for other spectra.

The strength of acid sites over these samples was analyzed via NH_3_ temperature-programmed desorption (NH_3_-TPD) using a Micromeritics AutoChem II 2920 apparatus (Microtrac, Japan) equipped with a thermal conductivity detector (TCD). Before the test, the samples were pretreated under an Ar atmosphere at 120 °C for 2 h and then cooled to 50 °C. Then, a 5% NH_3_/Ar mixture was introduced into the reactor for adsorption. After reaching an adsorption equilibrium, the gas was switched to He and passed through the samples while heating to 500 °C at a ramping rate of 10 °C min^−1^. The signals were recorded by TCD and a mass spectrometer (MS). In addition, the type and relative content of acid sites over these samples were titrated using pyridine with Fourier transform-infrared spectroscopy (FT-IR). The samples were pretreated at 120 °C for 2 h in a dynamic vacuum environment and then cooled to 50 °C to collect the background spectrum. After exposure to pyridine for 5 min, infrared curves were collected. Then, the samples were heated to 150 °C and 250 °C under a vacuum atmosphere to collect the infrared signals. The peaks at 1450 cm^−1^ and 1540 cm^−1^ were attributed to L and B acid sites, respectively [22].

## 3. Results and Discussion

### 3.1. Catalytic Performance of Cellulose Conversion into LA

**Figure 1:**
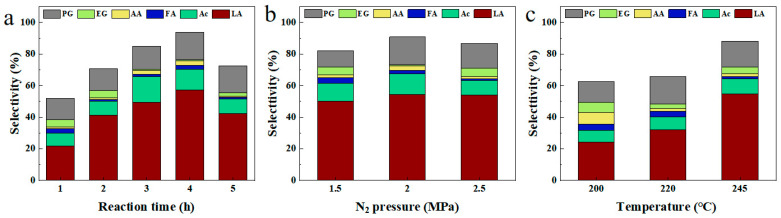
Catalytic performance of Au/W-ZnO-2 under different measurements of (**a**) Time, (**b**) Pressure, and (**c**) Temperature. PG: propylene glycol, EG: ethylene glycol, AA: acetic acid, FA: formic acid, Ac: acetone alcohol, LA: lactic acid.

The effects of reaction conditions on cellulose conversion to LA were systematically investigated using Au/W-ZnO-2 as the catalyst to optimize the reaction conditions (Table S1). First, the reaction time varied under the conditions of 2 MPa N_2_ and 245 °C. It was observed that after 1 h of the reaction time, cellulose was almost entirely converted, but the selectivity of products underwent significant changes as time increased. The main products identified were acetone alcohol, lactic acid, and 1,2-propanediol glycol (1,2-PG). The selectivity of acetone alcohol and LA showed an initial increasing trend followed by a decrease, with maximum selectivity at 3 and 4 h of the reaction time, respectively (Figure 1a). This suggests the possibility of a reaction path for the conversion of acetone alcohol to lactic acid, followed by conversion to alcohol.

Furthermore, pressure had little effect on the selectivity of LA, with changes within 3% for a range of 1.5–2.5 MPa N_2_ (Figure 1b). This finding implies that N_2_ was only utilized as a protective atmosphere and did not affect the conversion path of cellulose [23,24]. Temperature analyses indicated that cellulose could not be fully hydrolyzed at 220 °C, and the selectivity of LA was low. However, raising the reaction temperature to 245 °C led to complete cellulose transformation and improved LA selectivity, reaching 54.6% (Figure 1c). According to previous studies, lactic acid was further converted into alcohol at higher temperatures [25]. However, it should be noted that higher temperatures were not used for the experiments due to equipment limitations.

**Figure 2:**
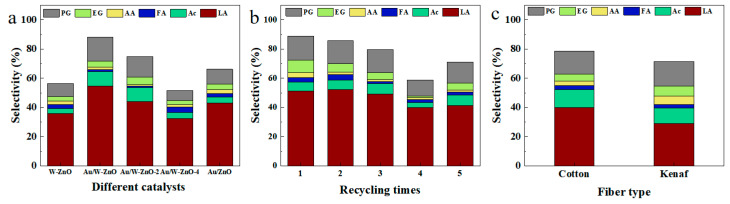
(**a**) Catalytic performance of various catalysts, (**b**) Recycle experiments over Au/W-ZnO. (**c**) Conversions of real biomass over Au/W-ZnO. Reaction conditions: 100 mg reactant, 50 mg catalyst, 2.0 MPa N_2_, 245 °C, 4 h. PG: propylene glycol, EG: ethylene glycol, AA: acetic acid, FA: formic acid, Ac: acetone alcohol, LA: lactic acid.

Under the optimal conditions obtained above, catalysts were evaluated for cellulose conversion. It was found that cellulose could be converted entirely over all catalysts, but the product distribution was significantly different (Figure 2a and Table 1). Amongst them, Au/W-ZnO demonstrated the highest LA selectivity of 54.6%, while W-ZnO and Au/ZnO had a LA selectivity of 35.7% and 43.1%, respectively. The partial removal of ZnO using hydrochloric acid decreased lactic acid’s selectivity to 41.0% and 32.3% on Au/W-ZnO-2 and Au/W-ZnO-4, respectively. These results suggest that the active metal Au, the metal W promoter, and the ZnO carrier all played a role in converting cellulose to lactic acid. Further characterization of the catalyst is needed to better understand each component’s roles.

On the premise of maintaining good activity and selectivity, the stability of the catalyst is crucial [26]. Therefore, recyclability was tested (Figure 2b and Tables S2 and S3). The results showed that Au/W-ZnO exhibited superior stability compared to other catalysts. The yield of LA remained at approximately 50% during the first three cycles and subsequently decreased to about 40% after the fourth and fifth cycles, respectively. However, the LA yield over the Au/W-ZnO-2 catalyst dropped from 44.0% to 32.4% after the initial cycle (Figure S1). The deficient performance and stability demonstrated that the ZnO support played a significant role in this process. On the one hand, the reduction in the ZnO content weakened its ability to convert glucose to fructose, ultimately leading to a decline in the rate of lactic acid formation. On the other hand, the lack of ZnO was not conducive to the stability of catalysts in the high-temperature hydrothermal environment, which made W leaching easier [27].

In addition, the catalyst requires a good conversion of real biomass as well. For example, Wang et al. used lead(II) ions to convert a sugar bagasse solution, obtaining a LA yield of 37% after 24 h [28]. Xia et al. fabricated the Fe-Sn-Beta catalyst and obtained a LA yield of 33.9% from microalgae [29]. To assess the catalytic performance of Au/W-ZnO for natural lignocellulosic feedstocks, cotton, and kenaf fiber were used since their natural yields are very large. (Figure 2c and Table S4). The raw materials were ground to sizes smaller than 60 meshes before the tests. It was found that cellulose could be converted entirely. Still, the LA selectivity obtained from the raw materials was slightly lower than that of cellulose conversion (40.1% for cotton fiber and 29.1% for kenaf fiber). This discrepancy might be due to lignin, hemicellulose, and other substances in the raw materials that could generate phenolic compounds, further inhibiting LA production [30]. The results showed that the catalyst has great application potential.

### 3.2. Catalyst Characterization

#### 3.2.1. Microstructure of Catalysts

**Figure 3:**
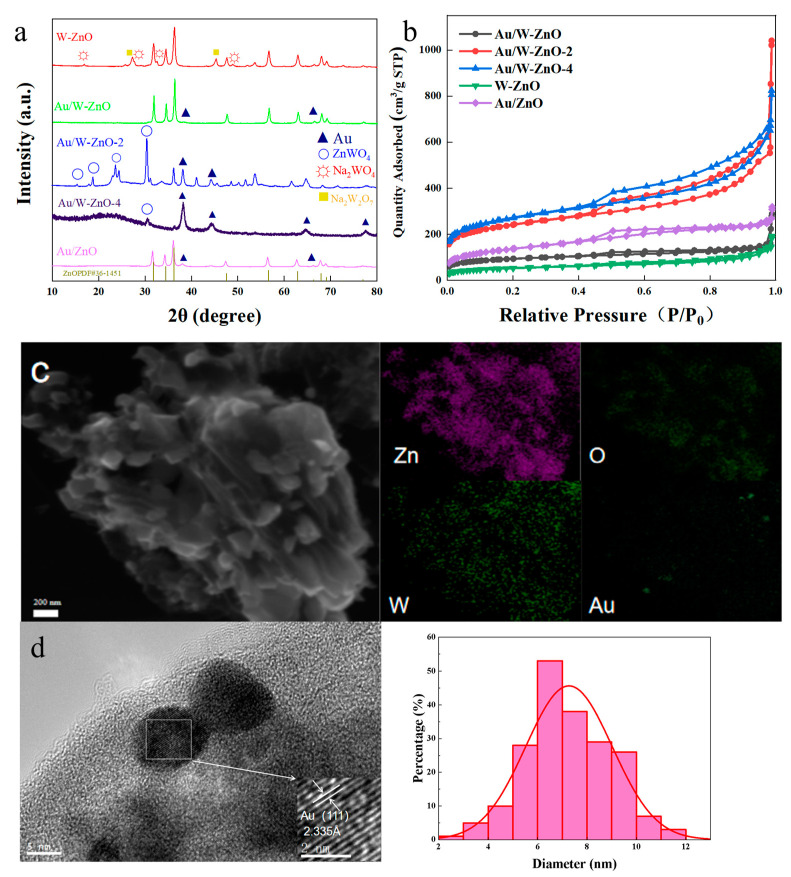
(**a**) XRD patterns of various catalysts, (**b**) N_2_ adsorption–desorption isotherm, (**c**) SEM images and EDS mappings of Au/W-ZnO, (**d**) TEM images and particle size distributions of Au/W-ZnO.

The elemental contents of the catalysts were examined using ICP analysis. The Au content was approximately 2.0 wt%, as listed in Table 2. The Zn content of W-ZnO, Au/W-ZnO, and Au/ZnO was around 57.0 wt%. However, after 2 and 4 h of acid treatment, the Zn content decreased to 1.4 and 0.6 wt%, respectively. The XRD analysis (Figure 3a) revealed the presence of ZnO diffraction peaks at 31.7°, 34.4°, and 36.3° for W-ZnO, and Au diffraction peaks at 38.1° and 44.4° when Au was loaded [31]. Furthermore, no diffraction peaks belonging to tungsten species were observed, suggesting that tungsten species were highly dispersed in the catalyst. However, after acid treatment, ZnO diffraction peaks decreased, and the ZnWO_4_ peak intensity increased for Au/W-ZnO-2 and Au/W-ZnO-4. This indicates that ZnO and W species in W-ZnO dissolved in the acidification process, and the resulting Zn^2+^ combined with tungstic acid to form ZnWO_4_, destroying the support’s structure. This is consistent with the decreased Zn content indicated by ICP. Moreover, the diffraction peak intensity of Au was the smallest over Au/W-ZnO. The average Au particle size analyzed by the Scherrer formula was 11.0 nm (Au/W-ZnO) but increased to 16.5 nm (Au/W-ZnO-2), 23.9 nm (Au/W-ZnO-4), and 14.2 nm (Au/ZnO).

The specific surface area and pore volume of the catalyst were examined. The adsorption–desorption curves in Figure 3b reveal an H4 hysteresis, signifying an irregularly distributed pore structure. The results in Table S5 show that W-ZnO and Au/W-ZnO had a similar surface area. However, after treating the samples in the HCl, both the surface areas and pore volumes clearly increased for Au/W-ZnO-2 and Au/W-ZnO-4. It could be caused by the destruction of the ZnO particles in the acidic solution. The catalysts’ surface morphology and elemental distribution were also studied (Figure 3c and Figure S2). The Au/ZnO catalyst demonstrated a uniform blocky structure, with some smaller round particles scattered on the surface. EDS further confirmed that the round particles corresponded to Au. By contrast, the Au/W-ZnO catalyst exhibited small lumps, likely resulting from the splintering of the blocky zinc oxide into smaller pieces while loading the W promoter in the sodium tungstate solution. Additionally, Au/W-ZnO-2 and Au/W-ZnO-4 transitioned from small to lamellar, and the EDS results indicated a decrease in the Zn element content. These phenomena were consistent with an increase in the specific surface area and abundance of pores. Subsequently, TEM analysis (Figure 3d and Figure S3) was used to determine the size of Au particles. It was approximately 7 nm over Au/W-ZnO but increased slightly to 8 nm over Au/W-ZnO-2 and Au/W-ZnO-4. This was likely due to the excessive destruction of the ZnO structure, leading to Au particle aggregation. When analyzing Au particle lattice spacing, the primarily exposed crystal planes were Au (111) and Au (200), which agreed with the XRD results. The higher dispersion of Au over Au/W-ZnO could provide more reaction sites and enhance the channeling of intermediates and products, ultimately boosting the reaction rate [32].

#### 3.2.2. Electronic Properties of Catalysts

**Figure 4:**
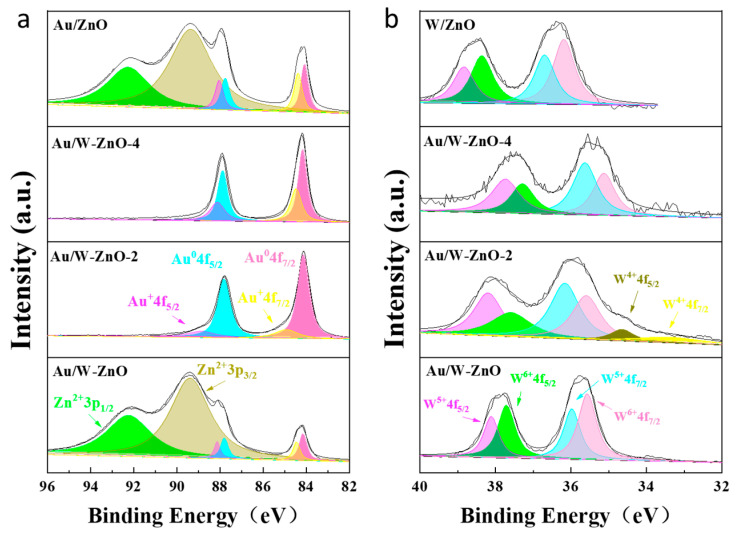
(**a**) Au 4f and (**b**) W 4f XPS spectra of catalysts. Green is Zn^2+^3p_1/2_, grey is Zn^2+^3p_3/2_, purple is Au^+^4f_5/2_, blue is Au^0^4f_5/2_, yellow is Au^+^4f_7/2_, pink is Au^0^4f_7/2_ in the (**a**). Purple is W^5+^4f_5/2_, green is W^6+^4f_5/2_, blue is W^5+^4f_7/2_, pink is W^6+^4f_7/2_, grey is W^4+^4f_5/2_, yellow is W^4+^4f_7/2_ in the (**b**).

X-ray photoelectron spectroscopy (XPS) was employed to scrutinize the chemical states of these catalysts, as presented in Figure 4. These findings indicate that Zn species are present on the surfaces of Au/ZnO and Au/W-ZnO in the form of Zn^2+^. Conversely, only some surface Zn species were identified on Au/W-ZnO-2 and Au/W-ZnO-4. The Au 4f spectrum exhibited the existence of metallic Au^0^ and Au^δ+^ on Au/ZnO, with the latter being Au species located at Au-ZnO interfaces [33]. Similarly, metallic Au^0^ and Au^δ+^ were detected on Au/W-ZnO, but the ratio of Au^δ+^/Au^0^ was higher than that of Au/ZnO. This suggested the occurrence of electron transfer between Au species and W species. The W 4f spectrum of W-ZnO was analyzed, and it was found that there were W^5+^ and W^6+^ species present. The two peaks at 37.7 eV and 35.5 eV corresponded to W^6+^ 4f_5/2_ and W^6+^ 4f_7/2_, respectively, while the two peaks at 38.1 eV and 35.9 eV corresponded to W^5+^ 4f_5/2_ and W^5+^ 4f_7/2_ [34,35]. On the Au/W-ZnO catalyst, the W species also existed in W^5+^ and W^6+^. The ratio of W^5+^/W^6+^ on Au/W-ZnO was lower than W-ZnO, and the percentage of Au^δ+^/Au^0^ increased for Au/W-ZnO-2 and Au/W-ZnO-4. This indicated that electron transfers between Au and W species increased with the destruction of the ZnO structure [36,37].

Given that the acidity and type of the catalyst were closely linked to the W species, and the acid sites were the main active sites for cellulose hydrolysis and further conversion to lactic acid, any changes in the chemical state of Au and W could directly affect the catalytic effect of the catalyst. Thus, the acid sites of the catalysts were further examined to determine their impact on catalytic performance.

#### 3.2.3. Acidic Sites of Catalysts

**Figure 5:**
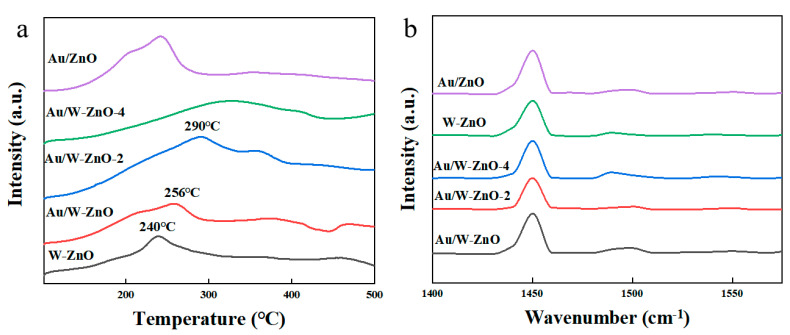
(**a**) NH_3_-TPD curves and (**b**) Py-IR spectra of the catalysts.

The strength of acid sites can be analyzed using NH_3_-TPD (Figure 5a). W-ZnO exhibited a broad desorption peak at 240 °C, corresponding to its weak acid sites. Two desorption peaks below 300 °C were observed on Au/ZnO, attributed to NH_3_ desorption from the ZnO and Au-ZnO interfaces. In addition to the expected NH_3_ desorption peaks on W-ZnO and Au/ZnO, a new desorption peak appeared at 350 °C on Au/W-ZnO, indicating the formation of moderately strong acid sites at the interface between Au and W species [16]. Moreover, after the destruction of the ZnO structure, the desorption peak at the high-temperature region remained. Its acidity increased, but the peaks at low temperatures almost disappeared. This suggests that acidity mainly originated from the presence of W and Au-W interfaces over Au/W-ZnO-2 and Au/W-ZnO-4, and the acidity caused by ZnO vanished due to the removal of ZnO.

The types of acidic sites can be further analyzed via pyridine-infrared spectroscopy (Py-IR) (Figure 5b). The results indicated that these catalysts mainly consisted of L acid sites and contained only a trace amount of B acid sites (Table S6). The Au/ZnO catalyst had 38.9 μmol g^−1^ L acid sites and 3.6 μmol g^−1^ B acid sites; the W-ZnO catalyst contained 43.2 μmol g^−1^ L acid sites and 4.5 μmol g^−1^ B acid sites. It implies that both ZnO and W promoters contribute to L acid sites. In addition, the L acid sites of Au/W-ZnO increased to 49.5 μmol g^−1^, but the number of B acid sites remained unchanged, suggesting the formation of a Au-O(OH)-W bond. Notably, the presence of a Au-O(OH)-W bond increased the amount of L acid sites and facilitated the stabilization of Au particles and W promoters. In addition, the L acid sites of Au/W-ZnO-2 and Au/W-ZnO-4 gradually decreased with the destruction of the ZnO structure. When further observing the relationship between acid sites and cellulose conversion, it was found that the total acid content had a good linear relationship with the selectivity of LA (Figure S4). This means that both B and L acid sites participated in cellulose-to-lactic acid transformation.

### 3.3. Proposed Reaction Mechanism

**Figure 6:**
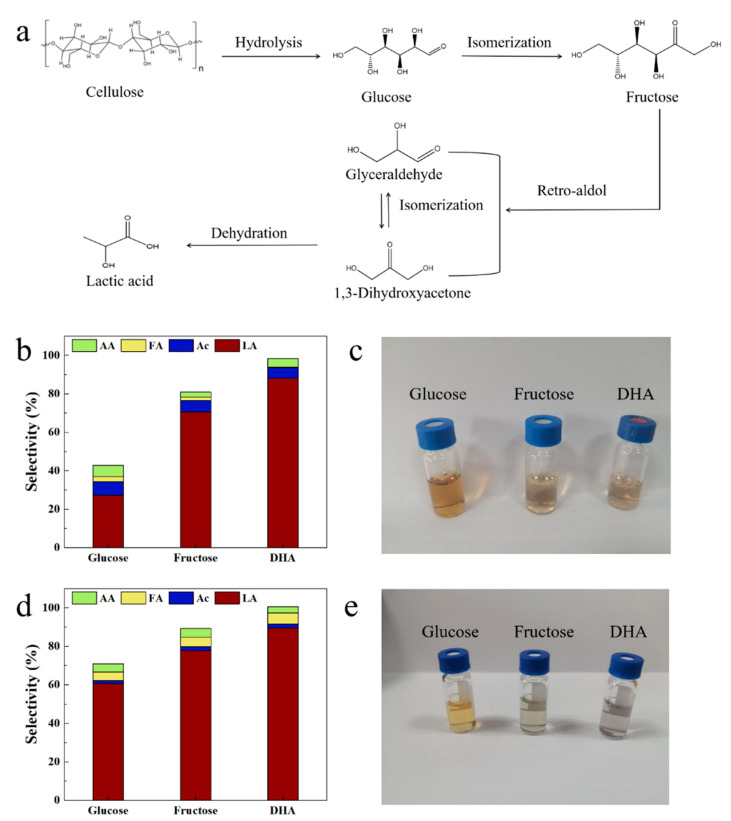
(**a**) Proposed catalytic mechanism of cellulose conversion into LA. Reaction results of different raw materials for (**b**) 15 min and (**d**) 4 h. The solution after reaction for (**c**) 15 min and (**e**) 4 h. PG: propylene glycol, EG: ethylene glycol, AA: acetic acid, FA: formic acid, Ac: acetone alcohol, LA: lactic acid.

Based on the above results, a possible route of cellulose conversion into LA is proposed (Figure 6a). First, cellulose was hydrolyzed to glucose at the B acid sites in a hydrothermal environment, which was then isomerized to fructose at the L acid sites. Fructose was further converted into 1,3-dihydroxyacetone (DHA) and propionaldehyde (PLY) through reverse aldol condensation, and DHA was finally converted into LA through isomerization [38].

To determine the decisive step in converting cellulose to LA over the Au/W-ZnO catalyst, experiments using glucose, fructose, and DHA as substrates were conducted (Figure 6b–e). The results show that the LA yield from fructose changed little after 15 min and 4 h of reactions, about 70.5% and 77.8%, respectively. A similar phenomenon occurred for DHA, about 88.1% and 89.5%, after 15 min and 4 h of reactions. However, using glucose as the substrate, the LA yield was 27.2% after 15 min of reaction but increased to 60.7%, prolonging the reaction time to 4 h. In addition, the solution after the glucose conversion was yellowish-brown. At the same time, the former two were colorless and transparent, indicating an incomplete conversion process. These results suggest that the transformation of fructose and DHA was faster and more efficient than the conversion of glucose. This meant that glucose conversion is the rate-limiting step in the overall conversion process.

## 4. Conclusions

In summary, this study demonstrates that the Au/W-ZnO catalyst exhibits excellent catalytic activity for converting cellulose to lactic acid, with a yield of 54.6% under the reaction conditions of 2 MPa N_2_, 245 °C, and 4 h. The catalysts also showed good stability and effectively converted different types of lignocellulosic feedstocks, such as cotton and kenaf fiber. Our findings suggest that the ZnO support provided abundant L acid sites and enhanced the stability of the catalyst, while W promoters increased the mesoporous structure and acid sites, and the Au metal enhanced the acidic sites through Au-O(OH)-W bonds. Furthermore, both B and L acid sites played critical roles in the cellulose conversion process. Notably, this study identified the isomerization of glucose to fructose as the rate-limiting step in the conversion of cellulose to lactic acid. This work provides valuable insights into designing highly efficient catalysts for converting cellulose to lactic acid, which has important implications for developing sustainable and renewable bio-based chemicals.

## Figures and Tables

**Table 1:** Catalytic performance of various catalysts.

Catalysts	Conversion (%)	Yield Based on Carbon (%)					
		LA	Ac	FA	AA	EG	PG
W-ZnO	>99	35.7	3.4	2.7	2.4	3.2	9.0
Au/W-ZnO	>99	54.6	9.8	1.4	1.8	4.0	16.5
Au/W-ZnO-2	>99	44.0	9.7	0.8	1.2	5.2	13.9
Au/W-ZnO-4	>99	32.3	4.3	3.6	1.9	2.6	7.0
Au/ZnO	>99	43.1	4.0	2.3	2.8	3.9	10.0

Reaction conditions: 100 mg reactant, 50 mg catalyst, 2.0 MPa N_2_, 245 °C, 4 h. PG: propylene glycol, EG: ethylene glycol, AA: acetic acid, FA: formic acid, Ac: acetone alcohol, LA: lactic acid.

**Table 2:** Elemental contents over the catalysts.

Catalysts	Au (wt%)	W (wt%)	Zn (wt%)
W-ZnO	/	4.4	57.0
Au/W-ZnO	2.3	1.6	60.6
Au/W-ZnO-2	1.7	1.7	1.4
Au/W-ZnO-4	2.3	1.6	0.6
Au/ZnO	2.0	/	56.3

## Data Availability

All of the data reported in this paper and Supplementary Materials are available online.

## Supplementary Materials

The following supporting information can be downloaded at: https://www.mdpi.com/article/10.3390/polym15214235/s1. Figure S1: Recycling experiments over Au/W-ZnO-2; Figure S2: SEM images; Figure S3: TEM images and particle size distributions of Au/W-ZnO-2 and Au/W-ZnO-4; Figure S4: Relationship between LA yield and total acid con-tent; Table S1: Catalytic performance of Au/W-ZnO under different reaction conditions; Table S2: Product distribution for the recycling test of Au/W-ZnO catalyst; Table S3: Product distribution for the recycling test of Au/W-ZnO-2 catalyst; Table S4: Composition of cotton and kenaf fiber; Table S5: Specific surface area and pore volume of different catalysts; Table S6: Acid contents of the catalysts.
