# Supplementary material for: Sustainable Production of Lactic Acid from Cellulose Using Au/W-ZnO Catalysts
Source: Polymers (Basel). 2023 Oct 26;15(21):4235. doi: 10.3390/polym15214235 (PMC10647521; doi:10.3390/polym15214235)
Supplement: Supplementary file 1 [file polymers-15-04235-s001.zip › polymers-2676504-supplementary.pdf]

polymers

MDPI

Supplementary Materials

Sustainable Production of Lactic Acid from Cellulose Using Au/W-ZnO Catalysts

Mingyu Guo, Chengfeng Zhou, Yuandong Cui, Wei Jiang, Guangting Han, Zhan Jiang *, Haoxi Ben * and Xiaoli Yang *

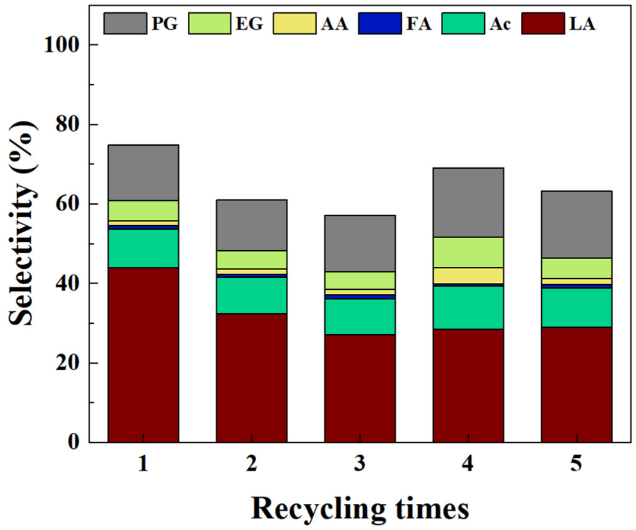

Figure S1. Recycling experiments over Au/W-ZnO-2. Reaction conditions: 100 mg reactant, 50 mg catalyst, 2.0 MPa N_2, 245 °C, 4 h. PG: propylene glycol, EG: ethylene glycol, AA: acetic acid, FA: formic acid, Ac: acetone alcohol, LA: lactic acid.

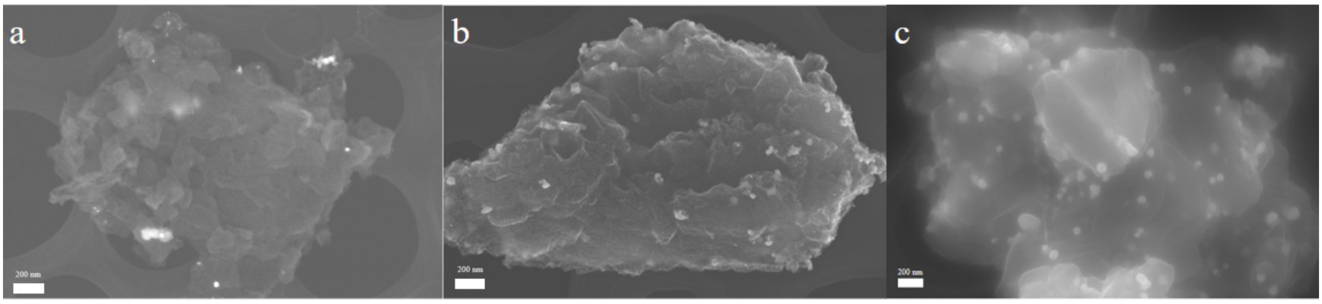

Figure S2. SEM images. a: Au/W-ZnO-2, b: Au/W-ZnO-4, c: Au/ZnO.

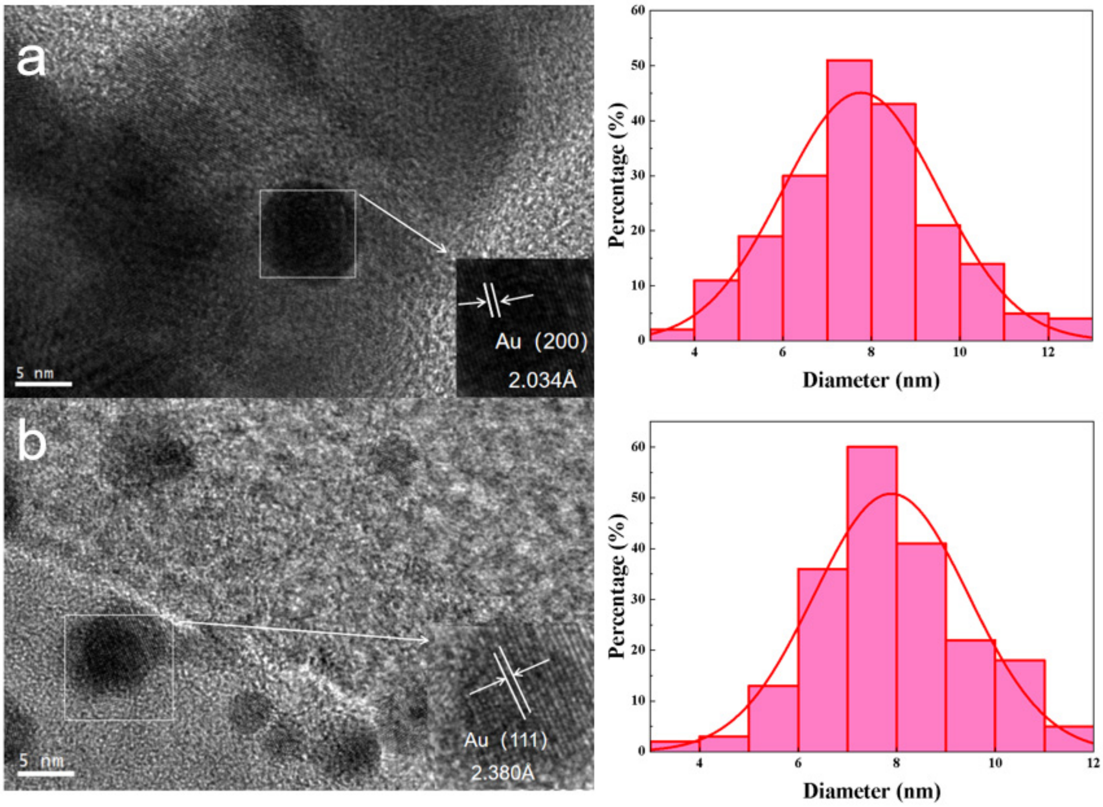

Figure S3. TEM images and particle size distributions of (a) Au/W-ZnO-2 and (b) Au/W-ZnO-4.

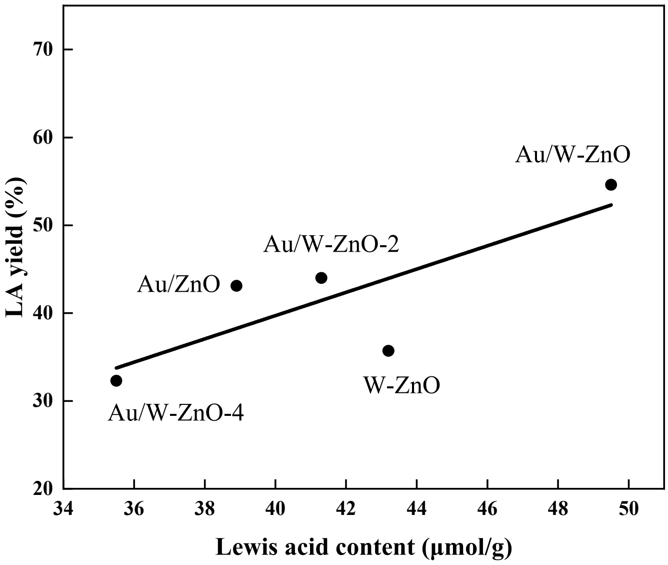

Figure S4. Relationship between LA yield and total acid content.

Table S1. Catalytic performance of Au/W-ZnO under different reaction conditions.

P (MPa)	T (°C)	Time (h)	Conversion (%)	Yield based on carbon (%)					
				LA	Ac	FA	AA	EG	PG
2	245	1	>99	21.7	8.4	2.7	1.2	4.4	13.6
2	245	2	>99	41.1	8.9	1.0	1.0	4.4	14.0
2	245	3	>99	49.3	16.5	1.3	2.4	0.9	14.5
2	245	4	>99	54.6	9.8	1.4	1.8	4.0	16.5
2	245	5	>99	42.4	9.1	1.0	1.0	2.0	17.0
1.5	245	4	>99	50.1	11.2	3.7	1.9	4.8	10.4
2.5	245	4	>99	53.9	10.3	0.8	1.3	3.1	17.8
2	200	4	>99	24.4	7.3	3.8	7.7	6.2	13.2
2	220	4	>99	32.2	8.2	3.4	1.8	3.0	17.3

PG: propylene glycol, EG: ethylene glycol, AA: acetic acid, FA: formic acid, Ac: acetone alcohol, LA: lactic acid.

Table S2. Product distribution for the recycling test of Au/W-ZnO catalyst.

Au/W-ZnO	Conversion (%)	Yield based on carbon (%)					
		LA	Ac	FA	AA	EG	PG
T1	>99	51.2	6.3	2.9	3.4	8.6	16.4
T2	>99	52.1	6.7	3.6	1.8	5.8	15.7
T3	>99	49.4	7.3	1.3	1.5	4.5	16.0
T4	>99	39.8	3.6	2.1	1.2	1.2	10.9
T5	>99	41.3	7.0	2.2	1.3	4.8	14.5

Reaction conditions: 2.0 MPa N_2, 245 °C, 4 h, 30 mL of H_2O, 100 mg of reactant, 50 mg of catalyst. PG: propylene glycol, EG: ethylene glycol, AA: acetic acid, FA: formic acid, Ac: acetone alcohol, LA: lactic acid.

Table S3. Product distribution for the recycling test of Au/W-ZnO-2 catalyst.

Au/W-ZnO-2	Conversion (%)	Yield based on carbon (%)					
		LA	Ac	FA	AA	EG	PG
T1	>99	44.0	9.7	0.8	1.2	5.2	13.9
T2	>99	32.4	9.1	0.7	1.5	4.6	12.7
T3	>99	27.0	9.1	1.0	1.4	4.4	14.2
T4	>99	28.5	10.8	0.6	4.1	7.7	17.4
T5	>99	29.0	9.9	0.8	1.5	5.1	16.9

Reaction conditions: 2.0 MPa N_2, 245 °C, 4 h, 30 mL of H_2O, 100 mg of reactant, 50 mg of catalyst. PG: propylene glycol, EG: ethylene glycol, AA: acetic acid, FA: formic acid, Ac: acetone alcohol, LA: lactic acid.

Table S4. Composition of cotton and kenaf fiber.

	Cellulose (%)	Hemicellulose (%)	Lignin (%)
Cotton	96.68±0.81	/	2.75±0.62
Kenaf	66.36±0.89	7.12±0.05	7.08±0.21

Table S5. Specific surface area and pore volume of different catalysts.

	Surface Area (m^2g^{-1})	Pore volume (cm^3g^{-1})
W-ZnO	282	0.02
Au/W-ZnO	332	0.06
Au/W-ZnO-2	852	0.13
Au/W-ZnO-4	960	0.14
Au/ZnO	477	0.03

Table S6. Acid contents of the catalysts.

	Lewis acid (μmol/g)	Bronsted acid (μmol/g)	Total acid content (μmol/g)
W-ZnO	43.2	4.5	47.7
Au/W-ZnO	49.5	4.7	54.2
Au/W-ZnO-2	41.3	2.0	43.3
Au/W-ZnO-4	35.5	5.4	40.9
Au/ZnO	38.9	3.6	42.5
